# Successful Treatment of Facial Acne Fulminans: Antimicrobial Agents and Oral Prednisolone as Promising Regimes

**DOI:** 10.1155/2017/7092910

**Published:** 2017-03-27

**Authors:** Amir Hossein Siadat, Anis Bostakian, Bahareh Abtahi-Naeini, Masoom Shahbazi

**Affiliations:** ^1^Skin Diseases and Leishmaniasis Research Center, School of Medicine, Isfahan University of Medical Sciences, Isfahan, Iran; ^2^Cancer Research Center, Semnan University of Medical Sciences, Semnan, Iran; ^3^Skin Diseases and Leishmaniasis Research Center, Department of Dermatology, Isfahan University of Medical Sciences, Isfahan, Iran

## Abstract

Acne fulminans (AF), also known as acne maligna, is a rare painful ulcerative form of acne with an abrupt onset and systemic symptoms. Its incidence appears to be decreasing, possibly because of earlier and better treatment of acne. This report highlights a case on a necrotizing facial wound due to AF that was successfully treated with oral prednisolone and antimicrobial medication.

## 1. Introduction

Acne fulminans (AF), also known as acne maligna, acute febrile ulcerative acne, and acute febrile ulcerative conglobate acne with polyarthralgia [1], is a serious variant of acne characterized by an abrupt onset of painful, inflammatory, ulcerative lesions covered with hemorrhagic crusts, which is accompanied by severe acne scarring. The lesions often appear on the upper chest and back [2, 3].

Systemic constitutional symptoms of AF include fluctuating fever, painful joints, malaise, loss of appetite, and laboratory abnormalities [4, 5]. The face is usually less severely involved than the trunk [5].

AF is a rare medical condition, with reports describing only approximately 150 cases. It needs to be treated immediately to avoid severe medical problems, such as permanent disfiguring scars [3]. Nowadays, fewer cases with this condition occur, possibly due to earlier and better acne treatment [3, 4].

Here we report a case with the abovementioned symptoms to highlight the need for early diagnosis and treatment of AF.

## 2. Case Presentation

**Figure 1:**
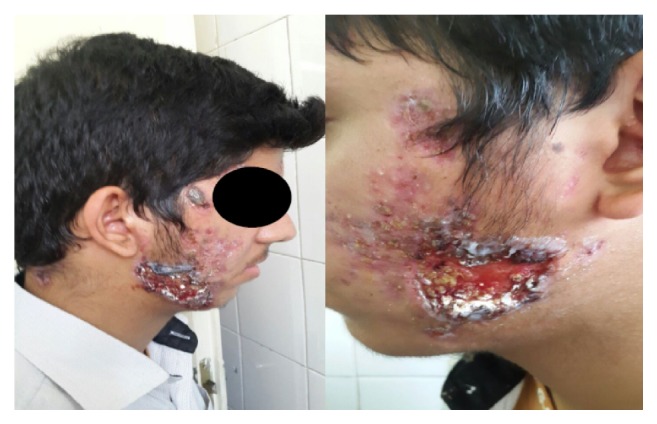
Acne fulminans. Hemorrhagic ulcerations with purulent crust scattered on both sides of the face.

A 15-year-old boy presented with a necrotizing bilateral facial wound on the jaw. He had been diagnosed with cystic acne on the face and upper back 3 months ago. He suddenly developed severe worsening of the acne lesions on the face and upper back, and nodular and ulcerated lesions appeared. Associated with the appearance of cutaneous lesions, he also reported fever, chills, arthralgia, and myalgia. On physical examination, he presented with pustules, nodules, and crusts on the face. Hemorrhagic ulcerations with purulent adherent crust were also present on both lateral jaws, as shown in Figure 1.

**Figure 2:**
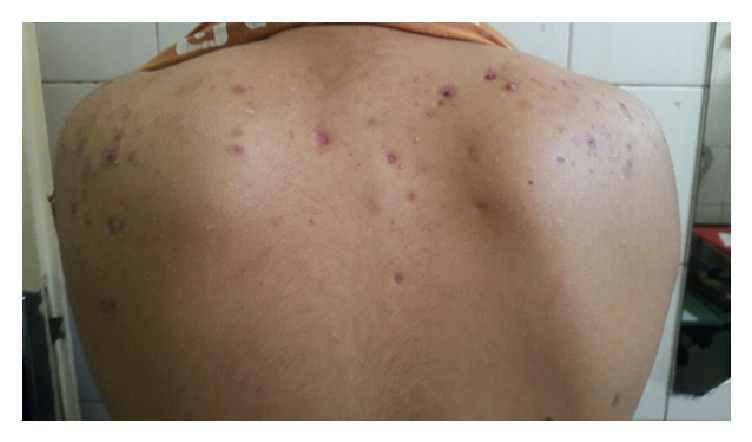
Some papules, pustules, small hemorrhagic crusts, and scar formation were scattered on the upper back of patient with acne fulminant.

Also he presented papules, pustules, and small hemorrhagic crusts which were scattered on the upper back and shoulders, as shown in Figure 2.

Physical examination of musculoskeletal system by an expert rheumatologist demonstrated no bony and joint involvement. There were not any palmoplantar pustulosis or psoriasis skin lesions.

Laboratory tests revealed abnormal white blood cell counts (16,000/mm3; segments 72.7%), hemoglobin (13.6 g/dL), erythrocyte sedimentation rate (75), serum ferritin level (178.8 ng/mL), and raised C-reactive protein levels.

Radiologic evaluation of axial skeleton and also sternoclavicular region revealed no sclerosis and osteolysis and periosteal reaction formation.

Magnetic resonance imaging (MRI) was also performed for detection of any occult bony involvement and the result was negative.

A biopsy specimen from the facial ulcer showed hyperkeratosis, acanthosis associated with follicular ostium destruction, and neutrophil infiltrations, which confirmed the diagnosis of AF. Treatment with broad-spectrum systemic antibiotics included 300 mg clindamycin thrice daily and 750 mg levofloxacin daily (according to the result of microbial colonization) in conjunction with 1 mg/kg/day oral prednisolone.

**Figure 3:**
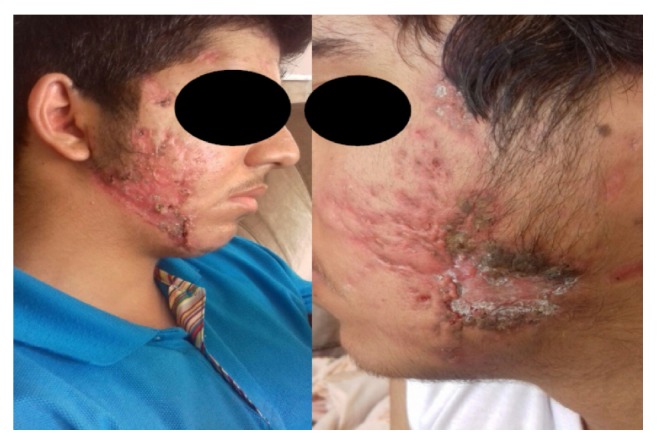
Acne fulminans after 28 days of oral prednisone and systemic antimicrobial regimen.

Primary control of the lesions was obtained over the course of 4 weeks with this therapeutic regimen, with noticeable decrease in the ulcerative lesions after treatment (Figure 3). Prednisolone was gradually reduced over a 2-month period, and a low dose of oral isotretinoin was initiated. The patient's acne fulminans gradually cleared within 3 months and healed, leaving a cicatricial scar on both lateral jaws.

## 3. Discussion

The etiology of AF is uncertain, but relationships with circulating androgens, infection, and explosive hypersensitivity reaction to surface bacteria have been postulated. Genetic and hereditary factors may also play an important role in the pathogenesis of AF in some patients [6, 7]. The term fulminans describes the sudden onset of the lesion and the course of this disease [3].

Patients with AF should immediately consult a dermatologist. The management of AF can be difficult; in addition to general supportive care, systemic corticosteroids, such as prednisone (20–60 mg/day), are the mainstay of therapy [8]. Other medications may be useful but usually require longer treatment periods. These medications include aspirin, dapsone [8], cyclosporine [2], antibiotic treatment probably due to their anti-inflammatory effect, and low-dose isotretinoin (after control of the acute phase of the disease) [4]. The data demonstrate that the treatment protocols that use a combination of prednisolone and isotretinoin lead to a faster control of systemic features as well as a faster clearance of the acne [9].

The use of antibiotics alone is ineffective but antibiotics can be effective if the microbial colonization or anti-inflammatory effects have been considered [8].

In our patient administration of antibiotic was based on the result of culture and microbial colonization.

Recurrences may be prevented by gradually reducing corticosteroids and possibly by the continuation or addition of systemic isotretinoin [10]. Although AF is usually identified because of its unique clinical features, the differential diagnosis should be considered especially at uncommon sites, including the face. Other potential diagnoses include rosacea fulminans [11], pyoderma gangrenosum, acne conglobate [8], and synovitis-acne-pustulosis-hyperostosis-osteitis (SAPHO) syndrome [12–14].

Although the clinical feature of severe rosacea fulminans can be similar to the acne fulminant, usually it is seen in women with higher age [11, 15]. Thus it is less probable for our patient.

Pyoderma gangrenosum characterized by painful progressive necrosis of the wound margins and the border of lesions typically is undermined and violaceous [16].

Acne conglobata rarely can affect the face but it has a more chronic course without a sudden onset [8].

SAPHO syndrome is suspected when a patient presents with a pustular skin disease in association with rheumatic pain but our patient had not any bony involvement and internal organs like the hematopoietic system [17].

Thus, clinicians should consider AF as a differential diagnosis for facial wounds as early diagnosis and treatment can reduce the associated morbidity.

## 4. Conclusion

AF should be suspected in patients presenting with an abrupt onset of a painful necrotizing facial wound of unknown etiology. In these settings antimicrobial agents combined with oral prednisone are an effective treatment for stabilizing clinical and laboratory parameters and preventing diseases progression.
